# Sirtuins, a promising target in slowing down the ageing process

**DOI:** 10.1007/s10522-017-9685-9

**Published:** 2017-03-03

**Authors:** Wioleta Grabowska, Ewa Sikora, Anna Bielak-Zmijewska

**Affiliations:** Laboratory of Molecular Bases of Aging, Department of Biochemistry, Nencki Institute of Experimental Biology of Polish Academy of Sciences, Pasteur Str. 3, 02-093 Warsaw, Poland

**Keywords:** Sirtuins, Ageing, Senescence, Curcumin

## Abstract

Ageing is a plastic process and can be successfully modulated by some biomedical approaches or pharmaceutics. In this manner it is possible to delay or even prevent some age-related pathologies. There are some defined interventions, which give promising results in animal models or even in human studies, resulting in lifespan elongation or healthspan improvement. One of the most promising targets for anti-ageing approaches are proteins belonging to the sirtuin family. Sirtuins were originally discovered as transcription repressors in yeast, however, nowadays they are known to occur in bacteria and eukaryotes (including mammals). In humans the family consists of seven members (SIRT1-7) that possess either mono-ADP ribosyltransferase or deacetylase activity. It is believed that sirtuins play key role during cell response to a variety of stresses, such as oxidative or genotoxic stress and are crucial for cell metabolism. Although some data put in question direct involvement of sirtuins in extending human lifespan, it was documented that proper lifestyle including physical activity and diet can influence healthspan via increasing the level of sirtuins. The search for an activator of sirtuins is one of the most extensive and robust topic of research. Some hopes are put on natural compounds, including curcumin. In this review we summarize the involvement and usefulness of sirtuins in anti-ageing interventions and discuss the potential role of curcumin in sirtuins regulation.

## Introduction

In the year 1979 a paper announcing discovery of mating-type regulator 1 (MAR1) in *Saccharomyces cerevisiae* was published (Klar et al. 1979). Lack of this protein resulted in the inhibition of silencing of HM loci, which control the mating type and sterility in yeast. Three more proteins with similar function were discovered later in 1979 and the nomenclature was unified thus creating a family of Sir (silent information regulator) proteins (Michan and Sinclair 2007). Shortly, it was shown that sirtuins are evolutionarily conserved from bacteria to humans (Vaquero 2009). We now know a number of processes sirtuins are involved in and we still discover their new functions. In bacteria phosphoribosyltransferases cobT and cobB catalyze the synthesis of the cobalamin biosynthetic intermediate (which transfers a ribose-phosphate moiety from nicotinic acid mononucleotide (NaMN) to dimethyl benzimidazole 2) and in archaea Sir-2-Af1 and Sir2-Af2 participate in transcription regulation (Tsang and Escalante-Semerena 1998). While in prokaryotes there are usually one or two sirtuin genes, eukaryotes can have multiple sirtuin genes. In yeast, in addition to the chief representative, Sir2, there are four more homologous proteins (Michan and Sinclair 2007). In mammals there are seven enzymes belonging to the sirtuin family, among which SIRT1 (silent information regulator T1) has the highest sequence homology to Sir2 in yeast and is the best studied family member. Modulation of sirtuin activity in mammals can regulate many processes such as gene expression, cell metabolism, apoptosis, DNA repair, cell cycle, development, immune response and neuroprotection (Michan and Sinclair 2007).

A significant rise in the interest in sirtuins occurred in 1999 when it was reported that Sir2 overexpression can extend yeast lifespan by as much as 70% (Kaeberlein et al. 1999). The anti-ageing action of sirtuins appears to be conserved from yeast to mammals, however the complexity of their function increases with the complexity of the organism. In yeast, the positive effect of sirtuins activity can be attributed to the increase in genomic stability in two ways. There are from 100 to 200 copies of ribosomal DNA (rDNA) in each yeast cell, however, only half of them are transcriptionally active, the rest remains silent (Sinclair and Guarente 1997). Together with other proteins, Sir2 participates in silencing of these regions. Such silencing prevents recombination between rDNA repeats and formation and accumulation of extrachromosomal rDNA circles (ERCs), which are a leading cause of yeast ageing (Sinclair and Guarente 1997). Mutations in Sir2 gene lead to accelerated accumulation of toxic ERCs, whereas Sir2 overexpression extends *S. cerevisiae* lifespan by silencing HML/R loci and inhibiting ERCs formation (Kaeberlein et al. 1999). Furthermore, along with yeast ageing Sir2 dissociates from HM loci, which results in termination of HM silencing and in sterility, which is a sign of yeast senescence (Sinclair and Guarente 1997). Therefore, changes in the localization of Sir2 result in epigenetic alterations that favor ageing. It was shown that Sir2 is indispensable for mediating positive effects of calorie restriction in yeast (Lin et al. 2000). It was also observed that the level of Sir2 increases during calorie restriction in *S. cerevisiae* (Bordone and Guarente 2005).

Further research revealed that sirtuin overexpression leads to lifespan extension also in other model organisms such as *Caenorhabditis elegans* and *Drosophila melanogaster*. In mammals sirtuins regulate numerous signaling pathways (not only those directly involved in ageing and senescence). This complex influence of sirtuins on mammalian ageing is discussed in this review.

### Function, structure and localization

In the early 1990s Braunstein et al. showed that regions silenced by Sir2 were characterized by reduced histone acetylation at the ε-amino group of N-terminal lysine residues (Braunstein et al. 1993). Some authors also observed that Sir2 overexpression in yeast led to global hypoacetylation. Soon it was discovered that the main activity of sirtuins is deacetylation of lysine residues. This is a two-step reaction—firstly sirtuins cleave nicotinamide adenine dinucleotide (NAD) to nicotinamide (NAM) and, subsequently, an acetyl/acyl group is transferred from the substrate to the ADP-ribose moiety of NAD; this results in the formation of 2′-*O*-acetyl-ADP-ribose and a deacetylated substrate (Tanner et al. 2000).

Sirtuins belong to class III histone deacetylases (HDAC). A distinguishing feature of this class is that the catalytic activity of the enzymes depends on NAD+ and is regulated by dynamic changes in NAD+ level and the NAD+/NADH ratio. Such requirement for NAD+ as a co-substrate suggests that sirtuins might have evolved as sensors of energy and redox status in the cell (Michan and Sinclair 2007). There are two pathways of NAD+ biosynthesis—de novo production and the so called salvage pathway. In the salvage pathway NAM is converted to nicotinamide mononucleotide (NMN) by nicotinamide phosphoribosyltransferase (NAMPT), a limiting enzyme for the whole pathway. Subsequently, NMN is converted to NAD+ by NMN/NaMN adenylyltransferase (NMNAT) (Chung et al. 2010). The level of NAMPT can influence sirtuin activity. NAD+ synthesis is coupled with the circadian/daily cycle due to the fact that NAMPT is regulated by a complex consisting of CLOCK (circadian locomotor output cycles kaput) and BMAL1 (brain and muscle aryl hydrocarbon receptor nuclear translocator-like 1) (Nakagawa and Guarente 2011). Unlike NAMPT, PARP1 activation by DNA damage results in a decrease in the NAD+ level (PARP1 uses NAD+ as a cofactor) and inhibition of sirtuin activity (Zhang 2003). NAM (another product of the reaction catalyzed by sirtuins) is a non-competitive inhibitor of sirtuin activity (Chung et al. 2010).

Sirtuins deacetylate not only histones but also some transcription factors and cytoplasmic proteins. Recent research shed some new light on sirtuins as it was shown that in addition to deacetylation they can remove some other moieties as well. For example, SIRT6 catalytic activity increases with the size of the aliphatic tail it removes, so that palmitoyl, myristoyl or butyryl are favored over acetyl moiety (Gertler and Cohen 2013). Therefore, it is now considered that sirtuins are not deacetylases but a more general term is proposed—deacylases (Jiang et al. 2013). Acetylation is a post-translational protein modification which can affect, among others, catalytic activity, stability and ability to bind to other proteins or chromatin (which is especially important in the case of histones).

In human we can distinguish seven sirtuins (SIRT1-7). Their catalytic domain consists of 275 amino acids and is common to all family members. Activity of some sirtuins is not limited only to protein deacetylation. ADP-ribosylation is the main activity for SIRT4, which lacks deacetylase activity, and is also characteristic for SIRT6 (Morris 2013). Moreover, SIRT5 can demalonylate and desuccinylate proteins (Du et al. 2011). SIRT1, SIRT6 and SIRT7 localize mainly in the nucleus. SIRT7 has been found to be a part of the RNA Pol I transcription machinery and is expressed in the nucleoli where it can bind to histones and positively regulate rDNA transcription (Ford et al. 2006). SIRT2 can be found mostly in the cytoplasm where its main substrate is α-tubulin (Li et al. 2007). Still, a fraction of SIRT2 can translocate to the nucleus where it takes part in regulation of the cell cycle (Dryden et al. 2003). SIRT3, SIRT4 and SIRT5 have been termed mitochondrial sirtuins. SIRT3 is cleaved to its active form by the mitochondrial matrix processing peptidase (Schwer et al. 2002). Full-length SIRT3 resides in the nucleus, however, in response to stress (such as DNA damage) it translocates to the mitochondria (Scher et al. 2007).

### Anti-ageing potential of sirtuins: in vivo and in vitro studies

Ageing is associated with numerous changes at the organismal, tissue as well as cellular level. With age, senescent cells accumulate in many tissues impairing their proper functioning. Senescent cells have a strong impact on surrounding cells. They modify the microenvironment by secreting certain cytokines, chemokines and mediators of inflammation. Such secretory phenotype is one of the causes of a low grade inflammation observed in old individuals and can induce senescence in neighboring cells as well as support tumor progression. Senescent cells, apart from the secretory phenotype, possess a set of features such as increased: level of cell cycle inhibitors, activity of senescence associated β-galactosidase, granularity and DNA damage. The elevation of DNA damage with age is the result of impaired efficiency of DNA repair systems. It is believed that DNA damage is the main cause of cellular senescence. It concerns both replicative (critically short telomeres are considered as DNA double strand breaks) and stress (oxidative, genotoxic) induced senescence. DNA damage is associated with normal functioning of cells and efficient repair systems are sufficient to protect cells from its accumulation. However, age-related decrease in the ability to repair DNA, causes increased damage accumulation and, in consequence, cell senescence. Sirtuins are indispensable for DNA repair, controlling inflammation and antioxidative defense which makes them good anti-senescence/anti-ageing targets.

Calorie restriction (CR) is so far the only effective way to extend lifespan without genetic or pharmacological intervention (more information about CR in the chapter concerning Intervention). The effects of calorie restriction (besides lifespan extension) are manifested by physiological and behavioral changes such as reduced size, decreased level of growth factors, glucose, triglycerides and increase in the locomotor and foraging activity (McCarter et al. 1997; Weed et al. 1997). The level of almost all sirtuins, except SIRT4, increases as an effect of calorie restriction (Watroba and Szukiewicz 2016). Therefore, it is believed that sirtuins mediate beneficial effects elicited by such diet. However, sirtuin anti-ageing activity is not limited to mediating the CR effects. Plethora of in vivo and in vitro studies show importance of these enzymes for reaching a lifespan characteristic for a particular species.

#### SIRT1

SIRT1 is the best studied in the family. It plays an important role during fetal development. In the case of mouse zygotes lacking both copies of SIRT1 gene only half of the expected individuals are born of which only 20% reach maturity. Such mice are sterile, smaller than normal individuals, develop more slowly and experience abnormalities in morphogenesis of the eye and heart. The latter likely contributes to the neonatal lethality of SIRT1 depleted mice (McBurney et al. 2003; Cheng et al. 2003). Additionally, among heterozygous embryos cases of anencephaly were reported.

The level of SIRT1 decreases in the liver with age, probably due to lower NAD+ availability (Braidy et al. 2011) while a simultaneous increase in accumulation of DNA damage occurs. Age-dependent decrease in the level of SIRT1 was observed also in the arteries, suggesting its involvement in the ageing of the cardiovascular system (Bai et al. 2014). Decrease in SIRT1, caused by accelerated senescence of cord blood endothelial cells, was also a cause of early vascular dysfunction observed in low birth weight preterm infants (Vassallo et al. 2014). SIRT1 deficiency promoted expression of genes characteristic for ageing (Hwang et al. 2013).

Mice with an extra copy of SIRT1 gene are characterized by a lower level of DNA damage and of p16, which are the hallmarks of ageing (Herranz et al. 2010). It was shown, that tissue-specific overexpression of SIRT1 in cardiac muscle cells diminished the area affected by myocardial infarction and facilitated recovery (Hsu et al. 2010). It was also shown that some single-nucleotide polymorphisms (SNP) in the SIRT1 gene could affect SIRT1 activity and correlate with BMI and a tendency to diet-induced obesity (Clark et al. 2012). However, no correlation between changes in SIRT1 activity (caused by SNP) and lifespan extension was found (Flachsbart et al. 2006).

SIRT1 was shown to delay replicative senescence of normal human umbilical cord fibroblasts and regulate both replicative and premature senescence in stem cells and differentiated cells exposed to oxidative stress (Bellizzi et al. 2005; Brown et al. 2013). Activation of the salvage pathway in vascular smooth muscle cells (VSMC) results in an increase in the replicative lifespan of these cells due to SIRT1 activation (Canto et al. 2009). Moreover, it was demonstrated that inhibition of NAMPT led to premature replicative senescence, while its overexpression delayed it (Yang and Sauve 2006). The level of SIRT1 decreases in tissues, in which cells proliferate during the organismal lifespan or during long term in vitro culture, as we have recently also shown for VSMC (Bielak-Zmijewska et al. 2014), but not in immortalized cells (Sasaki et al. 2006). In H_2_O_2_- or genotoxic stress-induced cellular senescence PARP1 becomes activated, which results in depletion of NAD+ resources and leads to a decrease in SIRT1 activity (Furukawa et al. 2007). There are data suggesting that SIRT1 can be involved in decision-making over cellular senescence or apoptosis. In the 3′UTR region of the SIRT1 transcript there is a HuR binding site. HuR is an RNA-binding protein, which can stabilize a transcript when bound. The level of HuR decreases dramatically during senescence (which can also be the cause of the decrease in SIRT1 level observed with ageing). In response to oxidative DNA damage HuR is phosphorylated by Chk2, which leads to its dissociation from SIRT1 mRNA. As a result, there is a decrease in the level of SIRT1 and cells become more prone to apoptosis (Abdelmohsen et al. 2007). It is possible that the described phenomenon is one of the mechanisms responsible for sustaining the balance between DNA repair, senescence and apoptosis. High level of DNA damage can activate Chk2, which leads to a decrease in SIRT1 level and moves the balance towards apoptosis (Bosch-Presegué and Vaquero 2011).

Pleiotropic activity of SIRT1 makes it an important marker of cellular senescence as well as some diseases such as cardiovascular and neurodegenerative diseases, diabetes or cancer (Nakagawa and Guarente 2011).

#### SIRT2

Expression of SIRT2 decreases in fat tissue of obese people (Krishnan et al. 2012). On the other hand, the level of SIRT2 increases in white fat tissue and kidneys of mice subjected to calorie restriction (Wang et al. 2007). Recent studies suggest that SIRT2 can serve as a cellular senescence marker. It was shown that the level of SIRT2 increased in senescent cells (regardless of whether the inducing factor was stress, oncogene or exhaustion of replicative potential) but not in quiescent cells or in cells that entered apoptosis (Anwar et al. 2016). At the same time, the authors excluded SIRT2 as an indispensable factor in senescence induction. This suggests that the increase in the level of SIRT2 is rather the effect of the changes occurring in cells during senescence, than the cause of senescence.

#### SIRT3

SIRT3 is the only sirtuin for which evidence exists that it can influence longevity in humans. It was shown that a certain polymorphism in SIRT3 gene can be found more often in long-lived people (Bellizzi et al. 2007, 2005). A variable number of tandem repeats in intron five enhancer region can affect activity of this enhancer. People carrying the allele with the least active enhancer were less likely to survive to an old age. Such variant was practically absent in men over 90 years old living in Italy (Bellizzi et al. 2005). However, studies of other larger populations did not confirm those findings, suggesting that SIRT3 influence on longevity is negligible or even nonexistent (Lescai et al. 2009; Rose et al. 2003).

Mice lacking SIRT3 are characterized by decreased oxygen consumption and simultaneous increase in reactive oxygen species (ROS) production as well as higher oxidative stress in muscle (Jing et al. 2011). Such observations were confirmed in cell culture—cells lacking SIRT3 had increased ROS level, which could induce DNA damage and activate HIF1α (Finley et al. 2011; Bell et al. 2011). SIRT3 activates enzymes, that play key roles during CR, such as 3-hydroxy-3-methyl-glutaryl-CoA synthase responsible for ketone formation (Shimazu et al. 2010) and long chain acyl-CoA dehydrogenase responsible for long-chain fatty acid oxidation (Hirschey et al. 2010).

#### SIRT1, SIRT2, SIRT3

Recent data have shown that the ageing protection mechanism involving sirtuins is quite universal and concerns also germ cells. The ageing of oocytes reduces the quality of metaphase II oocytes, which undergo time-dependent deterioration following ovulation. In mouse oocytes aged in vivo or in vitro the expression of SIRT1, SIRT2 and SIRT3 was dramatically reduced. On the other hand, it has been shown that prolonged expression of SIRT1, SIRT2 and SIRT3 reduced mouse oocyte ageing both in vitro and in vivo (Zhang et al. 2016a), which suggests a potential protective role of these enzymes against postovulatory ageing. SIRT1 and SIRT3 are the sensors and guardians of the redox state in oocytes, granulosa cells and early embryos and therefore play a crucial role in female fertility especially when oocyte ageing is concerned (reviewed in Tatone et al. 2015).

The age-dependent changes in sirtuin level could be used as a diagnostic tool. Serum sirtuins are considered as a novel noninvasive protein marker of frailty (Kumar et al. 2014a). Frailty is a complex clinical state described as a characteristic set of features among older patients. Diagnosis of frailty is often difficult because of subtle and subjective clinical features, especially at the early stage of the syndrome. To the features of frailty belong: sarcopenia, cognitive decline, abnormal functioning of immune and neuroendocrine systems, poor energy regulation (Clegg et al. 2013). Currently, there is no defined treatment for frailty. It will be useful to find a set of biochemical abnormalities associated with frailty for better and earlier diagnosis. Sirtuins circulating in serum could be potential markers of frailty. As suggested by analysis of people diagnosed as frail in comparison to non frail individuals, lower levels of SIRT1 and SIRT3 were associated with frailty.

#### SIRT6

The first evidence that sirtuins can be involved in regulation of mammalian ageing came from mice lacking SIRT6. It appears that among sirtuins, SIRT6 depletion exhibits the most severe phenotype as it seems to be indispensable for reaching a normal lifespan. Three weeks after birth such mice exhibit symptoms of degeneration and premature ageing such as sudden decrease in subcutaneous fat, lordokyphosis, colitis, severe lymphopenia, osteopenia, which all together result in death in about the fourth week of life. SIRT6^−/−^ mice are also smaller than wild type individuals. Furthermore, severe metabolic abnormalities were observed i.e. low level of IGF-1 and glucose (Mostoslavsky et al. 2006). Later, it was shown that the main reason of premature death was hypoglycemia caused by increased glucose uptake (due to higher expression of GLUT1 and GLUT 4 transporters) (Xiao et al. 2010; Zhong et al. 2010). On the other hand, Kanfi et al. demonstrated that overexpression of SIRT6 could also reduce the activity of the IGF-1 pathway. They observed a decrease in the level of IGF-1, the level of IGF-binding protein was increased, and the phosphorylation status of the main components of the IGF-1 signaling pathway was altered. Such changes facilitated glucose tolerance and reduced fat accumulation, which resulted in lifespan extension of male mice (Kanfi et al. 2012).

Mouse embryonal fibroblasts (MEF) and embryonal stem (ES) cells devoid of SIRT6 are characterized by decreased proliferation rate and increased genomic instability as well as sensitivity to stress manifested by chromosome fragmentation, detached centromeres, chromosome loss and translocations. SIRT6 level decreases in human fibroblasts during senescence (Sharma et al. 2013) but also in vascular smooth muscle cells and endothelial cells isolated from human aorta as we have recently demonstrated (Grabowska et al. 2016).

#### SIRT7

SIRT7^−/−^ mice age prematurely and are characterized by a progeroid phenotype and lethal heart hypertrophy (Vakhrusheva et al. 2008). During replicative senescence SIRT7 translocates from nucleoli to chromatin and cytoplasm (Grob et al. 2009), which can result in reduced rDNA transcription. Localization, activity, functions and role in senescence/ageing of all sirtuins are summarized in Table 1.

**Table 1:** Summary of the effects of various mammalian sirtuins, their localization, and intracellular targets

Sirtuin and localization	Enzymatic activity	Targets and substrates			Function	Tissue expression	Ageing and age-related diseases	
		Modification	Activation	Inhibition			Increase/involvement in CR	Decrease
SIRT1 nuclear/cytosolic	Deacetylase	H1, H3, H4, (H1K26, H1K9, H3K9, H3K56, H3K14, H4K16) α tubulin, p53-(stabilization)	Suv39h1, LKB1, AMPK, NBS1, XPA, Mn-SOD, WRN, Ku70	NFκB, p300, p66shc, mTOR	DNA repair, glucose metabolism, differentiation, neuroprotection, insulin secretion, vascular protection	Brain, adipose tissue, heart, kidney, liver, retina, skeletal muscle, vessels, uterus	Cell survival, longevity, physical activity/increase in CR	Cellular senescence, oxidative stress, inflammation, neurodegeneration, cardiovascular diseases, adiposity, insulin resistance, liver steatosis
			FOXO, PGC-1α					
SIRT2 cytosolic/nuclear	Deacetylase	α tubulin, H4K16	FOXO	NFκB, p53	Cell-cycle control (transition from G2 to M phase), adipose tissue development and functionality	Adipose tissue, brain, heart, kidney, liver, skeletal muscle, vessels	Longevity/increase in CR	Oxidative stress, neurodegeneration
SIRT3 mitochondrial/nuclear/cytosolic	Deacetylase	H3, H4 (H3K9, H4K16)	FOXO, Ku70, Mn-SOD, catalase, IDH2	p53, HIF1α	Regulation of mitochondrial metabolism, ATP production	Adipose tissue, brain, heart, kidney, liver, oocytes, skeletal muscle, vessels	Longevity, metabolic health, glucose homeostasis/increase in CR	Oxidative stress, neurodegeneration, cardiac hypertrophy, adiposity, liver steatosis
SIRT4 mitochondrial	ADP-ribosyl-transferase			GDH, AMPK	Insulin secretion, regulation of mitochondrial metabolism, DNA repair	Brain, heart, kidney, liver, vessels, pancreatic β-cells		Fatty acid oxidation
SIRT5 mitochondrial/cytosolic/nuclear	Deacetylase demalonylase desuccinylase		SOD1		Urea cycle	Brain, heart, kidney, liver, vessels, thymus, testis, skeletal muscle	Increase in CR	Oxidative stress, fatty acid oxidation
SIRT6 nuclear (associated with chromatin)	Deacetylase, ADP-ribosyl-transferase	H2B, H3 (H2BK12, H3K9, H3K56), WRN (stabilization)	FOXO, PARP1, CtIP	NFκB, IGF-1	DNA repair, telomere protection, genome stability, cholesterol homeostasis, regulation of glycolysis and gluconeogenesis	Brain, heart, kidney, liver, vessels, retina, skeletal muscle, thymus, testis, ovary	Longevity, glucose homeostasis/increase in CR	Cardiac hypertrophy, adiposity, liver steatosis, inflammation, insulin resistance
SIRT7 nucleolar/nuclear	Deacetylase	H2A, H2B, H3 (H3K18)	FOXO	RNA poly-merase I	Regulation of rRNA transcription, cell cycle regulation, cardioprotection	Heart, vessels, liver, brain, skeletal muscle, peripheral blood cells, spleen, testis	Increase in CR	Cardiac hypertrophy

*AMPK* AMP-dependent kinase, *CtIP* C-terminal binding protein interacting protein, *DNA-PKcs* DNA-dependent protein kinase catalytic subunit, *FOXO (FOXO3a, FOXO1)* Forkhead box “O” transcription factor, *GDH* glutamate dehydrogenase, *H1, H2A, H2B, H3, H4* histone; *HIF1α* hypoxia-inducible factor 1α, *IGF-1* insulin-like growth factor 1, *IDH2* isocitrate dehydrogenase 2, *LKB1* liver kinase B1, *Mn-SOD* manganese superoxide dismutase, *mTOR* mammalian target of rapamycin, *NBS1* Nijmegen breakage syndrome 1, *NFκB* nuclear factor κB, *PARP1* poly(ADP-ribose) polymerase 1, *PGC-1α* PPARγ coactivator1α, *SOD1* superoxide dismutase 1, *Suv39H1* suppressor of variegation 3–9 homolog 1, *WRN* Werner syndrome ATP-dependent helicase, *XPA* xeroderma pigmentosum group A

### The mechanisms of senescence modulation by sirtuins

The data presented above support the notion that sirtuins play an important role during ageing. It is best evidenced by a widely observed decrease in the level of almost all sirtuins in senescent cells. The mechanism of their action is very complex and not entirely understood yet.

During cellular senescence changes in chromatin condensation and gene expression occur. Such changes in chromatin structure can influence genome stability, making DNA more susceptible to damage, which is considered the main cause of senescence. Sirtuins play a vital role in sustaining genome integrity. They take part in maintaining normal chromatin condensation state, in DNA damage response and repair, modulate oxidative stress and energy metabolism. Let us take a closer look at the role of each sirtuin in these processes.

#### Influence on chromatin condensation and gene expression

Among cells isolated from mice lacking both copies of SIRT1 gene, almost 40% have impaired chromosome structure including breaks or relaxed/disorganized chromatin (in comparison to 5% in normal individuals) (Wang et al. 2008). It is suggested that such abnormalities can be the effect of an increase in the acetylation of H3K9, caused by lack of SIRT1. Acetylation of H3K9 prevents its trimethylation and impairs binding of heterochromatin protein 1 alpha (HP1α) responsible for keeping chromatin in a closed state (Wang et al. 2008). SIRT1 (and also other sirtuins), through histone deacetylation, takes part in formation of the constitutive as well as facultative heterochromatin. The removal of acyl groups from histones enhances their affinity to DNA and impedes the access of transcription factors to DNA resulting in silencing of genes neighboring the deacetylated histones (Michan and Sinclair 2007). SIRT1 preferentially deacetylates H4K16, H3K9, H3K56 and H1K26 (Poulose and Raju 2015) and also H1K9 and H3K14 during heterochromatin formation (Michan and Sinclair 2007). It was shown that SIRT1 can be found in telomere and pericentromere regions. Oxidative stress inhibits this interaction, which results in altered gene expression (Oberdoerffer et al. 2008; Palacios et al. 2010). Moreover, SIRT1 deficient mice lack pericentromeric heterochromatin foci (Bosch-Presegué et al. 2011), which suggest its involvement in formation of constitutive heterochromatin.

SIRT1 can influence chromatin condensation not only by deacetylating histones, but also by regulating histone expression and modulating the level and activity of some histone modifying enzymes (Vaquero et al. 2007). SIRT1 can inhibit Suv39h1 methyltransferase degradation by inhibiting polyubiquitination of this methyltransferase by MDM2. Moreover, deacetylation of K266 in the catalytic domain of Suv39h1 activates it (Vaquero et al. 2007). Therefore, SIRT1 promotes H3K9 trimethylation not only by deacetylation but also through cooperation with Suv39h1 (Bosch-Presegué and Vaquero 2011). Under oxidative stress, SIRT1 along with Suv39h1 and nucleomethylin initiate formation of facultative heterochromatin in the rDNA region. This, in turn, inhibits ribosome formation and decreases protein expression in general, which protects cells from energy deprivation-dependent apoptosis (Murayama et al. 2008) and, facilitates repair. Moreover, SIRT1 can deacetylate TBP [TATA-box-binding protein]-associated factor I 68 (TAF_I_68) impairing its DNA-binding activity, and in this way, inhibiting RNAPolI-dependent transcription of rDNA (Muth et al. 2001). In addition to Suv39h1, SIRT1 can modulate the activity of p300 histone acetyltransferase. SIRT1 inhibits p300 activity by deacetylating K1020 and K1024 (Bouras et al. 2005). In this way it contributes to the decreased level of histone acetylation.

SIRT2 participates in formation of metaphase chromosomes via H4K16 deacetylation (Vaquero et al. 2006). The level of SIRT2 fluctuates during cell cycle reaching its peak at the M phase and G2/M transition (Vaquero et al. 2006). Overexpression of SIRT2 can delay mitotic exit (Dryden et al. 2003).

SIRT3, as the main mitochondrial deacetylase, plays an important role in homeostasis of these organelles. Under stress the nuclear fraction of SIRT3 can deacetylate H4K16 and H3K9 regulating expression of genes involved in mitochondrial biogenesis and metabolism (Scher et al. 2007). Moreover, no hyperacetylation is observed in SIRT3^−/−^ cells, which suggests that SIRT3 is involved in regulation of only specific genes or regions (Scher et al. 2007).

**Fig. 1:**
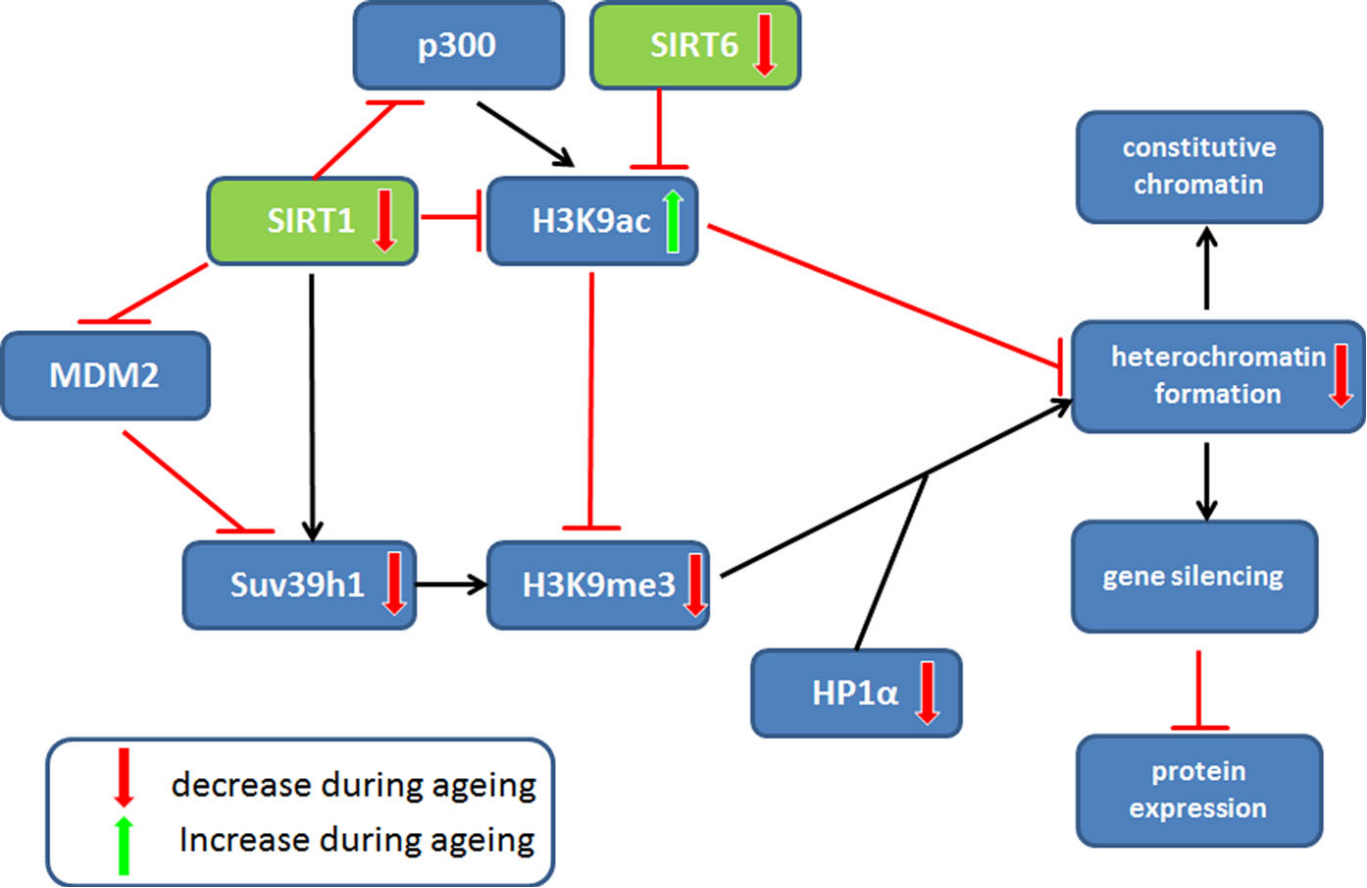
Role of SIRT1 and SIRT6 in chromatin condensation. SIRT1 and SIRT6 promote formation of heterochromatin in three ways. Firstly, both of the sirtuins deacetylate H3K9 enabling its trimethylation and subsequent binding of HP1α indispensable for heterochromatin formation. Secondly, SIRT1 decreases activity of p300 histone acetyltransferase. Lastly, SIRT1 activates Suv39h1 methyltransferase by deacetylating K266 in its catalytic domain. Moreover, SIRT1 inhibits polyubiquitination of Suv39h1 by MDM2 and prevents its degradation. *Arrows* indicate positive regulation. *Lines* with *T*-shaped ending indicate inhibition. *Thick upward* and *downward arrows* inside *boxes* indicate increase or decrease during aging, respectively. (Color figure online)

SIRT6 is a deacetylase as well as ADP-ribosylase acting mainly on histones. This sirtuin deacetylates H3K9 in the promotor regions of, among others, genes involved in metabolism (Zhong et al. 2010). In MEF and ES cells derived from SIRT6 knockout mice, H3K9 hyperacetylation in telomeres was observed. Such hyperacetylation caused a decrease in the level of trimethylated H3K9 in telomeres and chromatin relaxation in these regions. This suggests that SIRT6 can protect cells from telomere dysfunction (Cardus et al. 2013). In particular, SIRT6 deacetylates H3K9 in telomere regions in response to DNA damage (Gertler and Cohen 2013), which results in tightening and stabilization of the telomere structure. SIRT6 telomere binding is dynamic, and the strongest interaction is observed during the S phase of the cell cycle (Michishita et al. 2008). Moreover, SIRT6 stabilized ATP-dependent helicase WRN and prevented telomere dysfunction during DNA replication (Gertler and Cohen 2013). SIRT6 substrates also include H2BK12 and H3K56, increased acetylation level of the latter is associated with genomic instability (Jiang et al. 2013; Gertler and Cohen 2013). The role of SIRT1 and SIRT6 in chromatin condensation is presented in Fig. 1.

SIRT7 interacts with promoter as well as transcribed regions of rDNA genes. This sirtuin deacetylates histones, in particular H2A and H2B (Ford et al. 2006), however, its main substrate is H3K18 (Barber et al. 2012). Deacetylation of this histone is associated with repression of tumor suppressor genes. Therefore, SIRT7 can support cancer phenotype by inhibiting expression of tumor suppressors. However, it must be noted that SIRT7 is required only to sustain cancer phenotype and does not promote oncogenic transformation of normal cells (Barber et al. 2012; Kim et al. 2013).

#### Influence on DNA damage and DNA repair

Unrepairable DNA damage is believed to be one of the basic causes of cellular senescence (Sedelnikova et al. 2004). Already in yeast it was observed that Sir2 takes part in DNA repair. Changes in the localization of Sir2 occur not only during senescence but also as a result of DNA damage. Sir2 dissociates from HM loci and moves to the sites of DNA breaks (Oberdoerffer et al. 2008). This has two effects: firstly, it induces expression of HM genes (involved in DNA damage repair) and secondly, inhibits proliferation giving time for DNA repair to occur. Moreover, at sites of DNA breaks, Sir2 deacetylates histones and DNA damage response proteins that recruit proteins responsible for DNA damage repair (Oberdoerffer et al. 2008). The involvement of sirtuins in DNA damage recognition and repair has been also observed in more complex organisms.

Under normal conditions SIRT1 is bound to hundreds of gene promoters in the mouse genome. The binding pattern is disturbed as a result of genotoxic stress as SIRT1 moves to DNA damage sites where it plays an important role in the recruitment and activation of repair proteins (Chung et al. 2010). In cells derived from SIRT1 knockout mice, aside from chromosomal aberrations, impaired DNA damage repair was observed further proving that this sirtuin is involved in double helix repair (Wang et al. 2008). SIRT1 interacts directly with NBS1 and maintains it in a hypoacetylated state, which allows for phosphorylation of S343 that is necessary for efficient DNA damage repair response and activation of the S-phase checkpoint (Yuan et al. 2007). Acetylation of WRN promotes its translocation to the nucleus while subsequent deacetylation by SIRT1 increases its activity and efficiency of DNA damage repair by HR (Li et al. 2008). In response to DNA damage Ku70 is acetylated on multiple lysine residues. This facilitates dissociation of Ku70 from BAX, which results in translocation of the latter to the mitochondria and induction of apoptosis. SIRT1 deacetylates Ku70 sustaining its interaction with BAX. This, in turn, inhibits apoptosis and facilitates Ku70-dependent DNA damage repair (Bosch-Presegué and Vaquero 2011). A similar role was shown for SIRT3. SIRT1 is also involved in the repair of single strand DNA breaks via nucleotide excision repair (NER). UV radiation (NER is the main pathway responsible for repair of UV-induced breaks) stimulates interaction of SIRT1 with xeroderma pigmentosum group A (XPA)—one of the key factors in NER. XPA recognizes DNA damage and recruits proteins essential for the repair process. SIRT1 deacetylates XPA on K63 and K67 facilitating its interaction with RPA32 (which stabilizes single-stranded DNA) and DNA damage repair (Fan and Luo 2010). Additionally, SIRT1 overexpression in mice inhibits telomere erosion while its silencing accelerates telomere shortening (Palacios et al. 2010).

SIRT6 also plays a considerable role in DNA repair and maintenance of genomic stability by integrating signals of DNA damage with activation of repair enzymes (Mao et al. 2011). This sirtuin is involved in HR, non-homologous end-joining (NHEJ) as well as base excision repair (BER) (Mostoslavsky et al. 2006). SIRT6 poly-ADP-ribosylates proteins localized in the vicinity of DNA breaks promoting recruitment of repair enzymes (Gertler and Cohen 2013). Moreover, in response to DNA damage SIRT6 dynamically binds to chromatin and induces global decrease in H3K9 acetylation. In this way it stabilizes the binding to DNA of the catalytic subunit of DNA-dependent protein kinase (DNA-PKcs)—a key component of NHEJ that facilitates the access of repair enzymes to double strand breaks. In response to oxidative stress SIRT6 mono-ADP-ribosylates K521 of PARP-1 increasing its activity and facilitating DNA repair via NHEJ and HR (Beneke 2012). SIRT6 increases the activity of C-terminal binding protein interacting protein (CtIP)—an enzyme responsible for excision of damaged DNA fragments during HR. Under normal conditions CtIP is acetylated, however, after DNA damage SIRT6 deacetylates it on K432, K526 and K604 promoting resection of damaged fragments (Kaidi et al. 2010). It was shown that SIRT6 is also indispensable for BER to occur, however neither direct interaction with any of the components involved in this pathway nor co-localization on the damage site were proven (Mostoslavsky et al. 2006; Tennen and Chua 2011).

The negative feedback loop between DNA damage and NAD+ level may also contribute to cell senescence. DNA damage can induce a decrease in the level of NAD+ due to increased PARP1 activity, which requires NAD+ as a co-factor. Repeated or chronic DNA damage can result in substantial depletion of NAD+ and decrease in sirtuin activity. This in turn, can disrupt DNA damage repair (causing increase in the number of DNA breaks) and impair mitochondria function. The latter may result in increased ROS production and further DNA damage (Imai and Guarente 2014). Therefore, NAD+ level and sirtuin activity can provide an interface between DNA damage and mitochondria function and combine DNA damage theory with Harman’s mitochondrial theory of ageing.

Not only direct involvement in DNA repair is important, as in the case of SIRT1 or SIRT6, which can modify a variety of proteins engaged in repair of DNA damage. Glucose and glutamine metabolism is also relevant in this process. Glutamine is the main nitrogen donor, not only for protein, but also nucleotide synthesis. SIRT4 plays an important role in DNA damage response by regulating mitochondrial glutamine metabolism. During DNA damage response SIRT4 inhibits the transport of intermediates to the Krebs cycle so that the nitrogen atom from glutamine can be used for the synthesis of purine nucleotides crucial in DNA repair process (Jeong et al. 2013). SIRT4 is also a negative regulator of glutamate dehydrogenase (GDH), the first enzyme in glutamine metabolism (Haigis et al. 2006). Lack of SIRT4 disturbed DNA damage repair and promoted accumulation of the damage, while SIRT4 overexpression supported the removal of γH2AX foci (Jeong et al. 2013).

#### Influence on oxidative stress and energy metabolism

Among sirtuins the most important role in anti-oxidative defense is played by SIRT3. Deacetylation of mitochondrial complex I and III by SIRT3 results in an increase in the efficiency of electron transport, which prevents ROS production (Haigis et al. 2012). Loss of this sirtuin results in hyper-acetylation of the components of mitochondrial complex I and a decrease in its activity and in ATP level (Ahn et al. 2008). Sirtuins can counteract oxidative stress also by modulating antioxidant enzymes. SIRT1 can influence the level of manganese superoxide dismutase (MnSOD) via cooperation with FOXO transcription factors. Deacetylation of FOXO3a by SIRT1 leads to an increase in the level of MnSOD and catalase (Chung et al. 2010), while recruitment of SIRT1 to the promoter region of MnSOD gene along with FOXO1 is indispensable to co-activate expression of this antioxidant enzyme (Daitoku et al. 2004). Sirtuins modulate not only the level of antioxidant enzymes but also their activity. It was shown that deacetylation of isocitrate dehydrogenase (IDH2) and of MnSOD on K122 by SIRT3 increases activity of these enzymes (Bell et al. 2011). Moreover, activity of SOD1 increased after desuccinylation by SIRT5 (Lin et al. 2013).

### Interactions with other proteins involved in senescence

#### Interaction with p53

Regulation of SIRT1 and p53 activity is mutual and complicated. In response to stress SIRT1 deacetylates p53 on K320, K373 and K382 in the C-terminal regulatory domain. Deacetylation inhibits p53-dependent transcription and apoptosis, which facilitates DNA damage repair (Cheng et al. 2003). It was shown that SIRT1 colocalizes with p53 in PML nuclear bodies where it antagonizes PML-induced acetylation of p53 and inhibits premature senescence (Langley et al. 2002). SIRT1 can also regulate localization of p53. In mouse embryonic stem cells SIRT1 deacetylates p53 on K379 in response to oxidative stress, which prevents its translocation to the nucleus. This results in an increase in p53 level in the cytoplasm and in mitochondrial-dependent apoptosis (Han et al. 2008). However, in SIRT1 knockout animals, the effects associated with modulation of p53 activity are not observed. This inconsistency can be explained by a redundant action of sirtuins—SIRT2 and SIRT3 can also interact with p53 (see below). SIRT2 can inhibit the activity of p53. It is suggested that SIRT3 can act as a regulator in p53-dependent senescence by inhibiting p53 ability to promote cell cycle arrest and senescence (Li et al. 2010).

The sirtuins-p53 interaction is reciprocal and also p53 can influence the activity of these enzymes. In the promoter region of SIRT1 and SIRT2 there are two p53-binding sites (Bosch-Presegué and Vaquero 2011; Anwar et al. 2016). Moreover, 3′UTR fragment of SIRT1 mRNA has a miR-34a-responsive element. miR-34a is a small noncoding RNA that can inhibit expression of some proteins. Active p53 can induce expression of miR-34a. Therefore, increased activity of p53 increases the level of miR-34a, which inhibits SIRT1 translation. This interplay can in part explain the decrease in SIRT1, which we and others observed during cellular senescence (see Grabowska et al. 2015 and Grabowska et al. 2016). There is also indirect interplay between SIRT1 and p53 activity. Both proteins depend on NAD+ level since sirtuins require it as a co-factor while NAD+ can bind to p53 tetramers and affect their conformation thus preventing DNA binding (McLure et al. 2004). p53 can also positively regulate the level of SIRT6 (Kanfi et al. 2008).

#### Interaction with FOXO family

FOXO transcription factors are believed to promote longevity although the precise mechanism in not yet fully understood. However, it has been shown that they act as sensors of the insulin/IGF-1 signaling pathway, which is crucial for ageing and longevity (Martins et al. 2016), and regulate expression of key antioxidant enzymes such as MnSOD and catalase (see above). Activity of the FOXO family can be regulated by phosphorylation and acetylation. Acetylation of these factors facilitates phosphorylation and inactivation therefore decreases their ability to bind to DNA (Matsuzaki et al. 2005). Sirtuins can deacetylate some members of the FOXO family such as FOXO1, FOXO3a and FOXO4 whereby inducing their activity (Michan and Sinclair 2007). It has been shown that deacetylation of FOXO3a increases expression of proteins involved not only in protection against oxidative stress but also in DNA repair and cell cycle checkpoints (Michan and Sinclair 2007). SIRT2 was demonstrated to be the main deacetylase of cytoplasmic FOXO1 (Zhao et al. 2010). On the other hand, FOXO1 can regulate expression of SIRT1 by binding to its gene promoter region (Xiong et al. 2011), which creates an autoregulatory feedback loop regulating SIRT1 expression.

#### Interaction with NFκB

NFκB transcription factor was shown to regulate the process of ageing and it seems that its main role is to transactivate genes the products of which contribute to the senescence associated secretory phenotype (SASP). It was shown that SIRT1 (and also SIRT2) can inhibit NFκB signaling by deacetylating p65 (RELA) on K310, which modulates its ability to bind DNA and induces transcription of proteins involved in inflammation. In consequence, SIRT1 activity leads to a decrease in inflammation (Chung et al. 2010). SIRT6 can also inactivate NFκB by direct interaction with its RELA subunit. Such effect is followed by inhibition and destabilization of RELA binding at target gene promoters (Gertler and Cohen 2013), which can contribute to inhibition of apoptosis and senescence. Moreover, SIRT6 destabilizes binding of this transcription factor by deacetylating H3K9 in gene promoters of NFκB target genes (Gertler and Cohen 2013). On the other hand, NFκB can decrease the activity of SIRT1, in a similar way to p53, by modulating miR-34a expression (Kauppinen et al. 2013).

#### Interaction with AMPK

Many studies revealed that increased AMPK (AMP-activated protein kinase) activity can extend the lifespan of some model organisms. It was also shown that AMPK can regulate several signaling pathways involved in senescence and ageing such as those engaging p53, mTOR and NFκB (Salminen and Kaarniranta 2012). Moreover, AMPK can regulate cellular energy expenditure/status through modulation of NAD+ level, which suggests that this kinase may be involved in regulation of sirtuin activity. Both AMPK and SIRT1 are activated as a result of CR, have similar molecular targets and biological activities (Ruderman et al. 2010). Activation of AMPK elevates the level of NAD+ (among others through increase in the level and activity of NAMPT) thereby increasing SIRT1 activity (Canto et al. 2009). On the other hand, SIRT1 activation increases the activity of AMPK by LKB1 deacetylation on K48. Deacetylated LKB1 migrates from the nucleus to the cytoplasm, binds to STE20-related adaptor protein (STRAD) and mouse embryo scaffold protein (MO25). The latter interaction induces LKB1 kinase activity and AMPK phosphorylation (Wang et al. 2011). This creates a positive feedback loop. SIRT4, on the other hand, inhibits AMPK activity (Ho et al. 2013).

#### Interaction with P66shc

SIRT1 negatively regulates the expression of P66shc (Chen et al. 2013), one of the three isoforms of the ShcA family. This protein is involved in oxidative stress because it stimulates mitochondrial ROS generation, and downregulates antioxidant enzyme synthesis (Miyazawa and Tsuji 2014). It also controls the lifespan/longevity (reviewed in Kong et al. 2016). SIRT1 decreases both the P66shc level and oxidative stress intensity (Zhou et al. 2011) because it binds to the gene promoter of P66shc and deacetylates histone H3, which reduces the transcription rate. P66shc knockout mice had longer lifespan and enhanced resistance to oxidative stress and age-related pathologies (Berry et al. 2007; Vikram et al. 2014; Kumar et al. 2014b; Ma et al. 2014). Moreover, P66shc inhibits the activity of FOXO3a transcription factor (Miyazawa and Tsuji 2014). Ageing-initiated P66shc-mediated endothelial dysfunction was shown both in clinical trials and animal experiments, however, not in P66shc knockout mice (Francia et al. 2004). This suggested that P66shc knockout mice were protected from endothelial dysfunctions. Moreover, such mice had 30% longer lifespan than control ones. SIRT1 seems to be involved in this protection (Berry et al. 2007). It has been also observed that CR, which evokes an increase in sirtuin activity, could reduce P66shc level (Zhou et al. 2011).

### Doubts

Despite plethora of research documenting beneficial influence of sirtuins on ageing and longevity there are also some conflicting data. Some authors completely exclude sirtuin involvement in CR-induced lifespan extension (as it was shown that CR can extend lifespan of Sir2-deficient yeast) (Tsuchiya et al. 2006). Others state that the fact that Sir2 overexpression combined with CR resulted in greater lifespan extension than each intervention alone suggests that sirtuins do not mediate the positive effect of CR (Kaeberlein et al. 2004). There are studies implying that increased longevity of some model organisms (such as *D. melanogaster* and *C. elegans*) after sirtuin overexpression is due to a lack of genetic background standardization and incorrectly matched controls (Burnett et al. 2011). Some data also show that SIRT1 can promote replicative senescence. Mouse embryonic fibroblasts lacking SIRT1 are characterized by increased replicative potential under conditions of chronic sublethal stress (Chua et al. 2005).

One of the reasons for such contradictory data can be the context-dependency of sirtuins. Activity of these deacetylases depends on the tissue and/or experimental conditions e.g. the presence of stress. The impact of sirtuin level was emphasized in the study of Alcendor et al. (2007). It was shown that 2.5–7.5 fold increase in SIRT1 level in mouse heart prevented age-associated cardiac hypertrophy, apoptosis, cardiac dysfunction and expression of senescence markers such as p15INK4b, p19ARF, p53. On the other hand, 12.5 fold increase in SIRT1 level promoted cardiac hypertrophy, induced apoptosis and promoted cardiomyopathy. The authors suggested that the beneficial effects could be the consequence of oxidative stress modulation since low and moderate overexpression of SIRT1 protects against oxidative stress, by eliciting an increase in the level of antioxidant enzymes and proteins such as catalase, heat shock proteins (Hsp40, Hsp70 and Hsp90), telomere repeat binding factor 2 (TRF2) and telomere reverse transcriptase (TERT). On the other hand, high SIRT1 level increased oxidative stress. High level of NAD+ dependent deacetylase can deplete the pool of this vital PARP1 cofactor and in this way impair DNA repair and mitochondrial respiration followed by decreased ATP production. Importantly, mice with moderate SIRT1 overexpression did not exhibit extended lifespan, while high sirtuin level shortened the animal life expectancy to a half. There is also a discrepancy of opinions as to SIRT1 contribution to atherosclerosis progression. It seems that the role of SIRT1 in this process depends on the cellular/physiological context as there are reports suggesting its protective function, and those implying promotion of plaque formation (Watroba and Szukiewicz 2016).

### Intervention in organismal ageing by sirtuin regulation

It has been proven that ageing is an extremely plastic process and its modulation can be very efficient. Ageing can be accelerated, slowed down, and, in some cases, even stopped or reversed under certain experimental conditions (Fahy et al. 2010). Anti-ageing interventions delay and prevent age-related disease onset. They include behavioral, dietary and pharmacological approaches. Also, many protein targets and many drugs are being tested for their effects on healthspan and lifespan. The intervention strategies include: (1) dietary interventions mimicking chronic dietary restriction, (2) inhibition of the mTOR–S6K pathway, (3) inhibition of the GH/IGF1 axis and (4) drugs that activate AMPK or specific sirtuins (Longo et al. 2015). In fact, all of the mentioned approaches are related to sirtuins. These enzymes are involved in mimicking dietary restriction, as it has been shown, for example, for resveratrol. Furthermore, inhibition of the mTOR–S6K pathway is caused by AMPK, which is regulated by sirtuins. In turn, the SIRT1-p53 pathway has been described to antagonize IGF-1-induced premature cellular senescence (Tran et al. 2014). Therefore sirtuins are extensively studied in the context of their role in alleviating symptoms of ageing and age-related diseases (Houtkooper et al. 2012; Hall et al. 2013; Poulose and Raju 2015).

#### Dietary restriction

Dietary/caloric restriction (DR)/(CR) (the reduction of calorie intake without causing malnutrition) is the only known intervention able to increase the lifespan in many species, including yeast, fruit flies, nematodes, fish, rats, mice, hamsters and dogs (Weindruch 1996; Masoro 2005; Ingram and Roth 2015) and possibly even primates (Ingram et al. 2006; Colman et al. 2009). Much research has suggested that lifespan extension and healthspan improvement brought by caloric restriction are mediated by mechanisms involving sirtuins. For example, some of the effects of caloric restriction in flies, worms and mammals have been shown to be mediated by SIRT1 (Rogina and Helfand 2004; Tissenbaum and Guarente 2001; Chen et al. 2005; Boily et al. 2008). Diet-induced aortic stiffness, developed within 2 months in mice fed HFD (high fat diet), can be prevented by SIRT1 induction in VSMC (Fry et al. 2016). Reduction of arterial stiffness can be also achieved by overnight fasting in mice fed HFD for 2 or 8 months but not in mice lacking functional SIRT1 in VSMC. Similar effect was observed after SIRT1 overexpression or treatment with SIRT1 activators. DR was also shown to induce SIRT6, which delayed ageing by suppressing NFκB signaling in aged mice after 6-month treatment or in cells cultured in low glucose condition (resistance to cellular senescence) (Zhang et al. 2016b). Dietary restriction is one of the most promising strategies for increasing lifespan and healthspan also in humans (reviewed in Longo et al. 2015). In humans such interventions are effective in lowering the prevalence of age-related loss of function and protecting against age-related pathologies, as evidenced by changes in the level of markers for type 2 diabetes, hypertension, cardiovascular disease, cancer, and dementia (Cava and Fontana 2013). Because long lasting DR is not recommended for most people and could be associated with undesirable side effects, less drastic dietary interventions should be considered and therefore drugs or supplements, which mimic the effects of DR are searched for. A promising strategy, potentially useful for humans, could be short-term fasting that could mimic DR. Because sirtuins can mediate many of the beneficial effects of DR (Satoh et al. 2013), therefore activators of the sirtuin pathway are very attractive candidates considered to mimic DR. To such compounds belongs resveratrol, the role of which in DR is well recognized and described (Chung et al. 2012), and probably also curcumin as has recently been shown by us (Grabowska et al. 2016).

#### mTOR inhibition

Inhibitors of the mTOR signaling are the major candidates for targeted interventions. This signaling pathway has been linked to lifespan and healthspan extension in model organisms (Johnson et al. 2013) because reduced mTOR signaling benefited both these phenomena. The best recognized inhibitor of mTOR is rapamycin, although a long term treatment can bring about some side effects (Hartford and Ratain 2007). S6 kinase (S6K) is a target of mTOR. Loss of S6K promoted longevity in yeast, flies, worms, and mice (Johnson et al. 2013). Sirtuins and AMPK are regulators of this kinase. It has been shown that increased SIRT1 activity resulting from resveratrol diet supplementation inhibited the mTOR/S6K pathway in mice (Liu et al. 2016).

#### Attenuation of IGF1/insulin signaling pathway

The IGF1/insulin signaling pathway is a very well recognized target in postponing ageing. In mammals upstream of IGF1 is a growth hormone (GH) (Brown-Borg and Bartke 2012). GH mutant mice (a reduction of plasma levels or disruption of the receptor) live 50% longer than wild-type ones. GH fulfills key metabolic functions, controls circulating IGF1 levels and acts also independently of IGF1. The insulin and IGF1 signaling pathway is strongly evolutionarily conserved. Both insulin and IGF are important in the maintenance of proper metabolism and organismal homeostasis. They control growth, development and regulate stress resistance. Activation of this pathway leads to phosphorylation of transcription factors belonging to the FOXO family. In turn, it has been shown that these transcription factors are required for impairing insulin/IGF-1 signaling to extend lifespan in worms (Kenyon et al. 1993; Melendez et al. 2003). Sirtuins are among the regulators of the transcriptional activity of FOXO proteins. Human IGF-1 receptor gene polymorphisms are associated with exceptional longevity (Suh et al. 2008) and low plasma IGF-1 concentrations predict further survival in long-lived people (Milman et al. 2014). Moreover, treatment with IGF-1 triggered premature cellular senescence (human primary IMR90 fibroblast and MEFs, mouse embryonic fibroblasts) in a p53-dependent manner and a recent study explained this result as being due to attenuation of SIRT1 functioning, followed by enhanced p53 acetylation and stabilization, and premature cellular senescence (Tran et al. 2014). DR is very effective in inhibiting insulin/IGF-1 signaling.

#### Dietary and pharmacological interventions

Functional foods and nutraceuticals/dietary ingredients are a great promise for health and longevity promotion and prevention of age-related chronic diseases (Ferrari 2004). The potent sirtuin-activating compounds (STACs) include several classes of plant-derived metabolites such as flavones, stilbenes, chalcones, and anthocyanidins, which directly activate SIRT1 in vitro. Several substances are reported to have anti-senescent effect in vitro by modulating the SIRT1 pathway. These compounds include a number of agents such as resveratrol (Kao et al. 2010), cilostazol (Ota et al. 2008), paeonol (Jamal et al. 2014), statins (Ota et al. 2010), hydrogen sulfide (Suo et al. 2013; Zheng et al. 2014) and persimmon (Lee et al. 2008). It is documented that polyphenols, to which curcumin also belongs, are able to modulate sirtuins (reviewed in Jayasena et al. 2013; Chung et al. 2010). The best recognized and described natural compound is resveratrol and there are a lot of papers summarizing its role in sirtuin stimulation on both the organismal and cellular level (Howitz et al. 2003; Ramis et al. 2015). Activation of SIRT1 by resveratrol supplementation led to increased lifespan and improved healthspan of several species i.e., mimicked the anti-ageing effect of DR (Baur et al. 2006; Mouchiroud et al. 2010). In human diploid fibroblasts resveratrol decreased or delayed cellular senescence (Huang et al. 2008). Other natural anti-ageing compounds are: quercetin, butein, fisetin, kaempferol, catechins and proanthocyanidins (reviewed in Jayasena et al. 2013). Several reports emphasized that dietary supplementation of polyphenols may protect against neurodegenerative, cardiovascular, inflammatory, metabolic diseases and cancer by enhancing SIRT1 deacetylase activity. However, in humans, the therapeutic and pharmacological potential of these natural compounds remains to be validated in clinical conditions. Their efficiency is, however, put into doubt because many natural compounds, including curcumin are bad leads for drugs (Baell and Walters 2014). However, polyphenols may act as prophylactic agents in terms of dietary intake rather than as therapeutic ones. Some natural compounds from Traditional Chinese Medicines (TCMs) are potent SIRT1 activators (Wang et al. 2016).

Another compound considered as anti-ageing one is melatonin. It is able to activate sirtuins and it has been observed that its level decreases with age (Ramis et al. 2015). It has been shown that melatonin prevents age-related alterations in apoptosis in dentate gyrus, which are associated with neurodegeneration, by increasing SIRT1 (Kireev et al. 2013). Adjudin, a derivative of lonidamine, an activator of SIRT3 (Bellizzi et al. 2005; Brown et al. 2013; Kincaid and Bossy-Wetzel 2013), is also considered as an anti-ageing factor (Xia and Geng 2016). Another compound that possesses anti-ageing function is icariin, an active ingredient of *Epimedium* in Berberidaceae (Lee et al. 1995). It is able to enhance the expression of SIRT6 (Chen et al. 2012). A polysaccharide derived from *Cornus officinalis* could slow down the progression of age-related cataracts by significantly increasing expression of SIRT1 mRNA and FOXO1 mRNA (Li et al. 2014). Oligonol, an antioxidant polyphenolic compound showing anti-inflammatory and anti-cancer properties, mainly found in lychee fruit, may act as an anti-ageing molecule by modulating the SIRT1/autophagy/AMPK pathway (Park et al. 2016). Spleen lymphocytes derived from old mice treated with oligonol showed increased cell proliferation. Moreover, this compound extended the lifespan of *C. elegans* infected with lethal Vibrio cholera (Park et al. 2016). Also, metformin, a herbal compound widely prescribed as oral hypoglycaemic drug for the treatment of type 2 diabetes, acts by SIRT1 activation (and FOXO1 elevation) in endothelial dysfunction caused by diabetes-related microvascular disease associated with accelerated endothelium senescence and ageing (Arunachalam et al. 2014).

Natural phytochemicals are effective sirtuin activators, but synthetic STACs, such as SRT1720, SRT2104, SRT1460, SRT2183, STAC-5, STAC-9, STAC-10 are considerably more potent, soluble, and bioavailable (Hubbard and Sinclair 2014; Minor et al. 2011). In preclinical models, STACs have shown effectiveness in treating age-related diseases and complications associated with ageing, including cancer, type 2 diabetes, inflammation, cardiovascular disease, stroke, and hepatic steatosis (Hubbard and Sinclair 2014). Based on mouse models, STACs could also be beneficial in neurodegeneration (Alzheimer’s or Parkinson’s disease) (Zhao et al. 2013; Graff et al. 2013; Hubbard and Sinclair 2014). SRT2104 extended both the mean and maximal lifespan of male mice fed a standard diet and this effect concurred with improved health, including enhanced motor coordination and decreased inflammation (Mercken et al. 2014).

An alternative approach to activating sirtuins is regulation of NAD + level by activating enzymes involved in biosynthesis of NAD or by inhibiting the CD38 NAD hydrolase (Wang et al. 2014; Escande et al. 2013; Braidy et al. 2014). Manipulation of the level of NAD+ leads to variations in the lifespan elongation effect of SIRT1. The compound that can antagonize nicotinamide inhibition of sirtuin deacetylating activity is isonicotinamide (Sauve et al. 2005). Inhibitors of NAM (natural inhibitor of sirtuin) exert the same effect as sirtuin activators (Sauve et al. 2005). Glucose restriction, which mimics DR, extended the lifespan of human Hs68 fibroblasts due to increased NAMPT expression, NAD+ level and sirtuin activity (Yang et al. 2015). In turn, lifespan extension was diminished by inhibition of NAMPT and sirtuins. Moreover, malate dehydrogenase, MDH1, which is involved in energy metabolism and reduces NAD+ to NADH during its catalytic reaction, plays also a critical role in cellular senescence. Its activity is reduced in human fibroblasts derived from elderly individuals and knock down of this enzyme in young fibroblast induces a senescence phenotype (Lee et al. 2012). Decrease in MDH1 and subsequent reduction in NAD/NADH ratio led to SIRT1 inhibition. Mice engineered to express additional copies of SIRT1 or SIRT6, or treated with STACs (resveratrol, SRT2104) or with NAD+ precursors, have improved organ function, physical endurance, disease resistance and longevity (Bonkowski and Sinclair 2016).

Activators of the AMPK pathway are considered as anti-ageing factors. SIRT1 increases the activity of AMPK through LKB1 activation, and, conversely, the activity of sirtuins is stimulated by AMPK. In turn, AMPK downregulates the mTOR pathway by inhibiting of S6K. To AMPK activators belong: 5-aminoimidazole-4-carboxamide riboside (AICAR), biguanides, salicylates, resveratrol, quercetin, catechins and, in certain range of concentrations, also curcumin (Coughlan et al. 2014; Grabowska et al. 2016).

Sirtuins are also responsible for epigenetic modifications (histone and non-histone proteins), which lead to changes in transcriptional activity of many genes. It is proposed that epigenetic factors contribute to ageing. Such factors are regulated by lifestyle, diet and exogenous stress. It is believed that epigenetic modifications (of both histones and DNA) have a comparable impact on gene expression to genetic modifications. It is suggested that manipulation of sirtuins could be beneficial for liefspan/healthspan modulation due to epigenetic changes. In humans, only nontoxic natural substances, such as curcumin or resveratrol, which could lead to histone deacetylation, should be considered for clinical testing as sirtuins activator. In general, functional food is a very promising element of anti-ageing intervention, including its potential influence on epigenetic modifications. Modulation of SIRT1 expression may represent a new means to counteract the effect of ageing.

#### Physical activity

Regular physical training is able to improve the quality of life. Exercise improves the resistance to oxidative stress, which could influence the pace of ageing and help maintaining the brain function (Marton et al. 2010). Extensive physical activity induces inflammation, increases ROS production and may impair the antioxidant defense system as it has been shown in skeletal muscle and blood (Banerjee et al. 2013). Mildly intense exercise can act as hormetin by eliciting a mild stress, which in turn activates defense mechanisms and brings beneficial effects including reduction of oxidative stress. Chronic exercise reduces oxidative stress by upregulating the activity of antioxidant enzymes (Greathouse et al. 2005). Mild physical activity is a potent activator of sirtuins (Csiszar et al. 2009; Radak et al. 2008). SIRT1 is suggested to be a master regulator of exercise-induced beneficial effects. It has been shown that long-term moderate exercise (36 weeks) induced increase in SIRT1 level in adult rat muscle, liver and heart (Bayod et al. 2012). Also, physical training promoted SIRT1 (as well as AMPK and FOXO3a) activity in muscle tissue in aged rat (Ferrara et al. 2008; Huang et al. 2016; Sahin et al. 2016). Similar effects were also described in humans (Bori et al. 2012). It has been demonstrated that in human skeletal muscle of both young and aged subjects, SIRT1 and AMPK gene expression increase after exercise. Exercise can at least partially recover the adaptive capacity to cope with mild oxidative stress that is lost in ageing and is the most effective intervention against several age-related pathologies such as sarcopenia, metabolic alterations (Pasini et al. 2012), neurodegeneration (Bayod et al. 2011; Mirochnic et al. 2009; Van Praag 2009) and cognitive loss (Kramer et al. 2006). Moderate forced exercise performed from an early age to adulthood has an important long-term impact on animal health. Exercise reduced plasma levels of glucose, cholesterol and triglycerides (Lalanza et al. 2012). In adult and older adult humans moderately intense exercise, for 30 min, 5 days a week, has beneficial effects (Colcombe and Kramer 2003; Rolland et al. 2010; Slentz et al. 2011). Exercise stimulates glucose uptake and mitochondrial biogenesis. Administration of AICAR is able to mimic the effect of physical activity (Hayashi et al. 1998; Song et al. 2002). Physical activity also elevated the level of NAMPT in human skeletal muscle (Costford et al. 2010). Even single bout of exercise increased SIRT1 expression in young individuals but such effect was not observed in old ones (Bori et al. 2012). Beneficial effect of exercise can be also observed at the cellular level. It has been shown that exercise inhibited replicative senescence of adipocytes (Schafer et al. 2016) and decreased the level of apoptosis in rat cardiomyocytes. With age, apoptotic pathway protein expression increases and the expression of the pro-survival p-Akt protein decreases significantly. Exercise increased activity of the IGF1R/PI3K/Akt survival pathway in the heart of young rats, however, in old animals the level of SIRT1 increased as a compensatory mechanism. Moreover, physical activity enhanced the SIRT1 longevity compensation pathway instead of elevating IGF1 survival signaling and in this manner improved cardiomyocyte survival (Lai et al. 2014). Physical activity is able to reduce the harmful effects of a fast food diet (FFD), prevent premature senescent cell accumulation and appearance of SASP in mice adipose tissue (Schafer et al. 2016). This suggests that exercise may provide restorative benefit by mitigating accrued senescent burden.

**Fig. 2:**
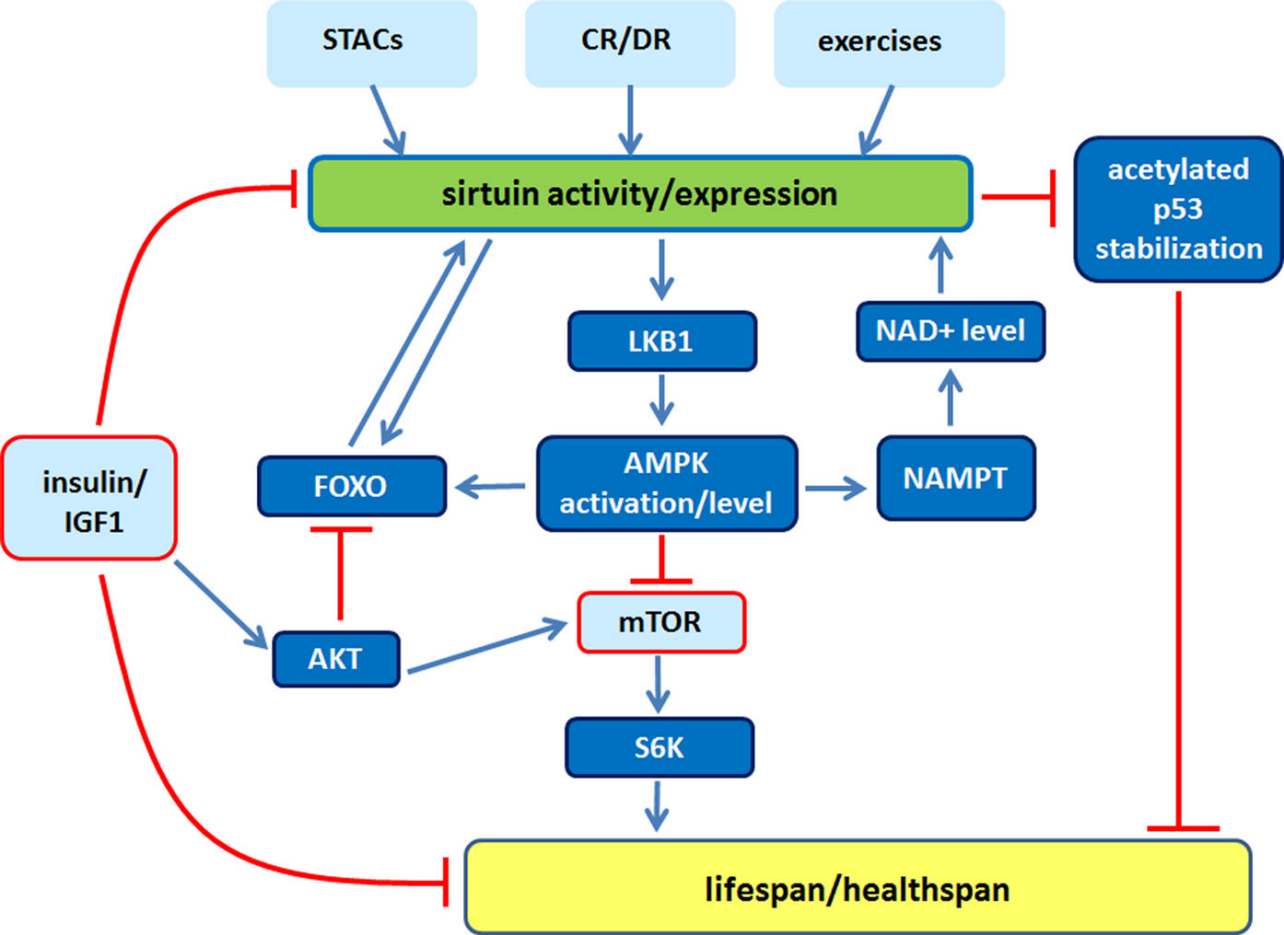
Involvement of sirtuins in lifespan/healthspan elongation pathways. Sirtuins modulate multiple pathways involved in mediating positive effects of some anti-ageing interventions, such as calorie/diet restriction (CR/DR) or exercise. Such effect can also be mimicked by sirtuin activating compounds (STACs). Prolonged activation of IGF1 pathway, involving PI3K-AKT, leads to phosphorylation and inhibition of FOXO and to inhibition of SIRT1 activity resulting in increased level of acetylated p53. Acetylation stabilizes p53, increases its activity and leads to premature cell senescence. Sirtuins contribute to life extension in animals with overactivated insulin/IGF1 signaling by increasing FOXO activity. Furthermore, sirtuins activate LKB1/AMPK pathway by deacetylating LKB1. AMPK downregulates mTOR/S6K activity preventing onset of senescence in cell cycle arrested cells. Moreover, AMPK can increase NAMPT activity, the enzyme indispensable in a salvage pathway, leading to NAD+ upregulation, which promotes sirtuin activity. *Arrows* indicate positive regulation. *Lines* with *T*-shaped ending indicate inhibition. Targets of lifespan/healthspan strategies are in *light color boxes*. *Light color boxes* with frame—pathways to be inhibited, without frame—beneficial activities. (Color figure online)

As mentioned above, sirtuin activation (by phytochemicals, CR, exercise, etc.) elicits an adaptive response to continuous mild exposures to stressors, in agreement with the hormesis principle (Bhakta-Guha and Efferth 2015). The involvement of sirtuins in lifespan/healthspan elongation strategies is summarized in Fig. 2.

### Curcumin in sirtuins regulation

Curcumin is a natural polyphenol extracted from a yellow pigment spice plant, turmeric, used for millennia in traditional medicine. Some polyphenols activate SIRT1 directly or indirectly, as has been shown in a variety of research models (Queen and Tollefsbol 2010). It has been proposed that curcumin possesses multiple biological properties including anti-oxidant, anti-inflammatory and anti-cancer activity, however there is also some rationale to consider this compound as an anti-ageing factor (Sandur et al. 2007; Sikora et al. 2010a, b; Salvioli et al. 2007). Curcumin was able to extend the lifespan of such organisms as fruit fly, nematodes and mice, and alleviated symptoms of some diseases including age-related ones (Liao et al. 2011). It reduced the impact of some harmful factors such as radiation or chemicals. Moreover, it increased the ability of cells to differentiate during replicative senescence as it was show in human epidermal keratinocytes (Berge et al. 2008). Curcumin possesses numerous target proteins and there are data showing that it is able to act by sirtuin activation. Several studies note that pretreatment with curcumin significantly enhances SIRT1 activation and attenuates oxidative stress (Sun et al. 2014; Yang et al. 2013). For example, pretreatment with curcumin attenuated mitochondrial oxidative damage induced by myocardial ischemia reperfusion injury through activation of SIRT1 (Yang et al. 2013). Likewise, curcumin blocked the neurotoxicity of amyloid-beta in rat cortical neurons by the same mechanism (Sun et al. 2014). The protective properties of curcumin, owed to the induction of sirtuins, help to reduce cisplatin chemotherapy-induced nephrotoxicity (Ugur et al. 2015) and protect kidney from gentamicin-induced acute kidney injury in animals (He et al. 2015). It has been shown that curcumin can elongate the lifespan of *Caenorhabditis elegans* but not when Sirt2 (the homolog of mammalian SIRT1) is mutated (Liao et al. 2011). Moreover, curcumin increased the level of SIRT1, which could help to prevent muscle damage (Sahin et al. 2016). Data concerning the impact of curcumin on cellular senescence are, however, confusing. On the one hand, it has been shown that curcumin attenuates hydrogen peroxide-induced premature senescence in HUVECs via activation of SIRT1 (Sun et al. 2015). Moreover, it was demonstrated that another curcuminoid, bisdemethoxycurcumin, could also antagonize the oxidative stress-induced premature senescence in WI38 fibroblasts through activation of the SIRT1/AMPK signaling pathway (Kitani et al. 2007). On the other hand, we showed that curcumin did not protect cells building the vasculature from premature senescence induced by DNA damaging agent, doxorubicin and did not postpone replicative senescence despite SIRT1 and AMPK upregulation (Grabowska et al. 2016). It is difficult to adjudicate whether curcumin can protect cells from senescence in vivo, but its role in sirtuin stimulation is convincing. Moreover, a lot of data show the reduction of symptoms of age-related diseases as a result of curcumin treatment. In particular, beneficial role of curcumin in the cardiovascular system is supported by numerous research data (Srivastava and Mehta 2009; Olszanecki et al. 2005; Yang et al. 2006). An animal study demonstrated that curcumin supplementation significantly ameliorated arterial dysfunction and oxidative stress associated with ageing (Fleenor et al. 2013). It seems justified to consider curcumin as a beneficial anti-pathological factor in the cardiovascular system. The neuroprotective role of curcumin is also mediated by SIRT1 induction, observed in primary cortical neurons in vitro. Accumulation of extracellular glutamate, the most abundant neurotransmitter in the brain involved in synaptic plasticity, learning, memory and other cognitive functions, can provoke neuronal injuries. Curcumin protected cortical neurons against glutamate excitotoxicity by SIRT1-mediated deacetylation of PGC-1α and preservation of mitochondrial functioning (Jia et al. 2016).

The effect of curcumin action strongly depends on its concentration. Curcumin belongs to hormetins, which means that at low concentration it may exert beneficial effects but is harmful at high concentrations (Calabrese 2014; Demirovic and Rattan 2011). Hormetins, by inducing a mild stress, and in consequence hormesis, are considered to be a promising strategy to slow down ageing and prevent or delay the onset of age-related diseases (Rattan 2012). The sensitivity to curcumin depends on cell type and probably the phase of the cell cycle. In vitro, in a certain range of concentrations, curcumin is toxic for all cell types, in another range inhibits the cell cycle and, at lower concentrations, seems to have no visible impact on cells (potentially beneficial doses according to the hormetic activity of curcumin). We showed that cytostatic doses of this factor induced cellular senescence in cancer cells (Mosieniak et al. 2012, 2016) and in cells building the vasculature (Grabowska et al. 2015). Curcumin-induced senescence of both vascular smooth muscle (VSMC) and endothelial (EC) cells was associated with decreased level of SIRT1 and SIRT6. Such downregulation seems to be characteristic for cell senescence not for curcumin. On the other hand, the level of mitochondrial SIRT3 and SIRT5 increased after curcumin treatment. These enzymes are stimulated in response to stress conditions and SIRT3, in particular, is an anti-oxidative protein which increases the activity of e.g. MnSOD. We postulate that activation of mitochondrial sirtuins is characteristic for dual curcumin action and could be considered as a protective mechanism induced by increased ROS production. Curcumin simultaneously increased ROS generation and activated proteins involved in anti-oxidative defense. This compound has also an impact on SIRT7. Downregulation of SIRT7 was observed at cytostatic concentration of curcumin. This could explain the arrest of the cell cycle, because it was documented that downregulation of SIRT7 may stop cell proliferation (Ford et al. 2006). Decreased activity of SIRT7 is associated with induction of nucleolar stress, which is related to inhibition of rDNA transcription (Lewinska et al. 2015). In turn, low doses of curcumin did not impair SIRT7 expression and even slightly increased its level (Grabowska et al. 2016). We tested also such concentrations of curcumin which have no impact on the proliferation of cells building the vasculature. We expected that such doses could delay the symptoms of cellular senescence, however, our results excluded this possibility, even though we observed that curcumin was able to increase sirtuin level, namely that of sirtuin 1, 3, 5, 6 and 7 (Grabowska et al. 2016). Therefore we concluded that curcumin anti-ageing activity is not due to delaying cellular senescence but is rather related to sirtuin elevation.

**Fig. 3:**
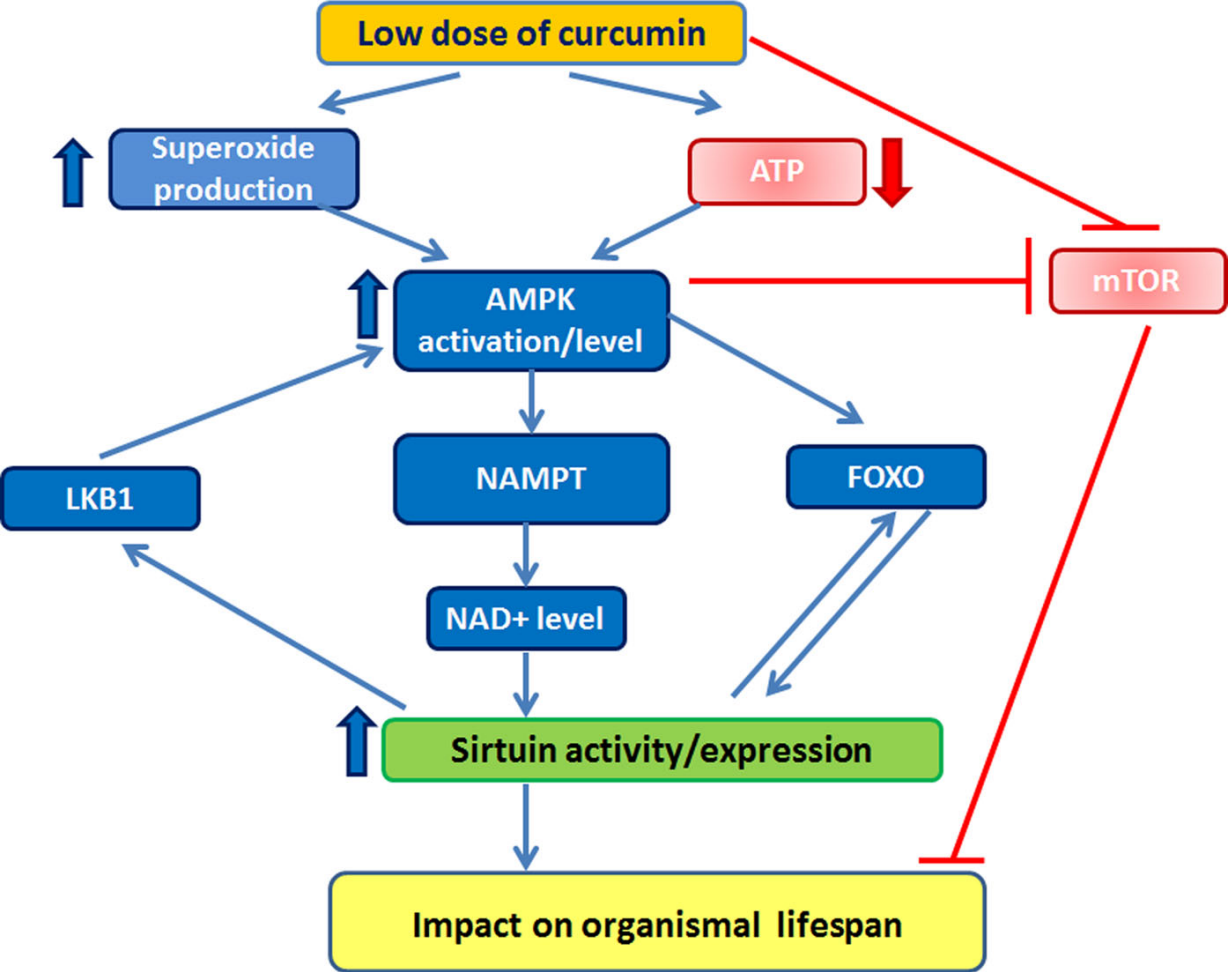
Mechanism of sirtuin activation by curcumin. We propose that curcumin increases sirtuins level and activity through upregulation and activation of AMPK. Such action can be a result of ATP reduction and initial increase in superoxide production (which is later neutralized by elevated expression of antioxidant enzymes). AMPK activation promotes NAD+ production via increase in NAMPT activity. Moreover, AMPK activates FOXO transcription factors which can induce sirtuin expression. Upregulation and activation of sirtuins promote LKB1/AMPK activity creating a positive feedback loop. Additionally, curcumin can contribute to postponing of ageing by inhibiting AKT/mTOR pathway. *Thin arrows* indicate positive regulation. *Lines* with *T*-shaped ending indicate inhibition. *Thick arrows* indicate decreasing or increasing level as described in Grabowska et al. (2016). The level/activity of proteins in *dark color boxes* increased upon curcumin supplementation, in *light color boxes*, decreased. (Color figure online)

It has been demonstrated that in senescence-accelerated mice a combination of resveratrol intake and habitual exercise is able to suppress the ageing-associated decline in physical performance (Murase et al. 2009). Resveratrol improves the effects of exercise in elderly rat hearts by enhancing FOXO3 phosphorylation via synergetic activation of SIRT1 and PI3K/Akt signaling (Lin et al. 2014). A similar effect was observed for curcumin supplementation. It has been documented that curcumin together with physical performance upregulates SIRT1 even more efficiently than dietary curcumin alone (Sahin et al. 2016). Curcumin supplementation affected the time of exhaustion in exercised rats. Moreover, curcumin treatment enhanced the effect of exercise and, together with exercise increased AMPK phosphorylation, NAD+/NADH ratio and SIRT1 expression in the muscle (Ray Hamidie et al. 2015). Improved exercise performance and fatigue prevention in mice was the result of increased resistance to stress conditions (Huang et al. 2015). Figure 3 summarizes the proposed mechanisms of sirtuin activation by curcumin.

Considering curcumin as a potential anti-ageing factor it is important to mention that it could act not only by mimicking of DR and exercise but is also able to inhibit the Akt/mTOR signaling pathway (Zhu et al. 2016; Guo et al. 2016; Jiao et al. 2016; Sikora et al. 2010a).

**Fig. 4:**
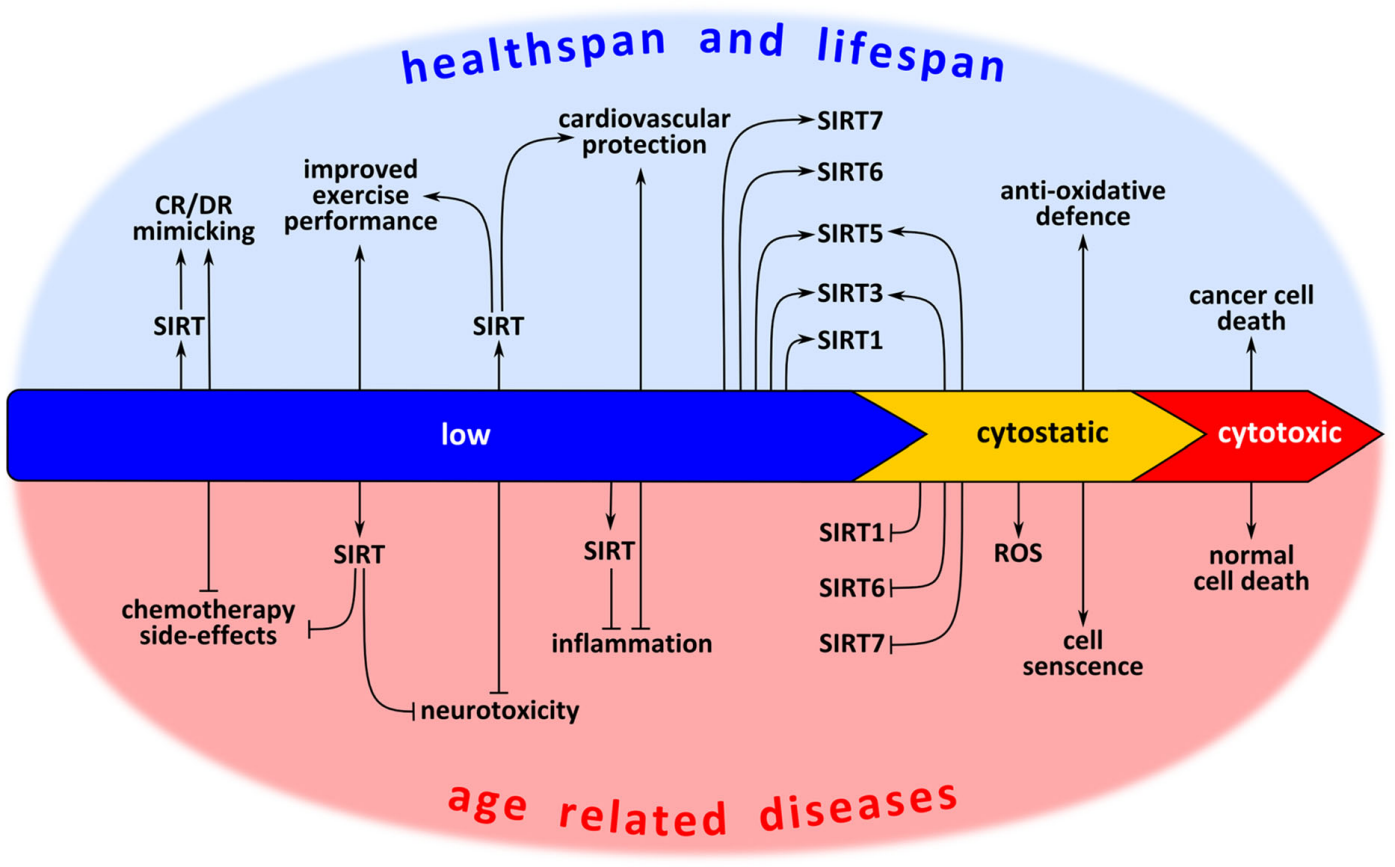
Dose-dependent activity of curcumin. Curcumin in high concentrations can be toxic while low concentrations may exert beneficial effects. In cytotoxic concentrations curcumin can be useful for eliminating cancer cells (a beneficial role), but may induce cell death in normal cells (a detrimental role). Cytostatic doses of curcumin induce senescence both in cancer and primary cells. In some situations this could be beneficial (senescence of cancer cells, protection from atherosclerosis), in others on the contrary (premature senescence of primary cells). Senescence upon curcumin treatment is associated with increased ROS production, upregulation of mitochondrial sirtuins (sirtuin 3 and 5), decrease in the level of sirtuins 1, 6 and 7 and upregulation of proteins involved in anti-oxidative defense. In turn, in low doses curcumin is able to upregulate the level of sirtuins. Animal studies show that supplementation of diet with curcumin can attenuate symptoms of some age-related diseases and improve exercise performance. Such effect is elicited via direct influence of curcumin on processes such as inflammation and/or indirectly via sirtuin upregulation and activation. *Arrows* indicate positive regulation. *Lines* with *T*-shaped ending indicate inhibition. Low, cytostatic and toxic refer to the range of curcumin concentrations

The impact of curcumin on lifespan/healthspan elongation strategies and protection from age-related pathologies is summarized in Fig. 4.

## Conclusions

Numerous data presented in the literature show sirtuins as a powerful tool in anti-ageing medicine/approach. Results from animal models, observations at the cellular level and data obtained from human studies suggest that sirtuins could be considered as a key regulator of ageing. The level of these enzymes decreases with age while their upregulation alleviates the symptoms of ageing/cellular senescence. Natural compounds present in the diet, classed as functional food/nutraceutics, could be an invaluable element of anti-ageing prophylactics or even intervention. Such compounds are nontoxic, easy to use and commonly available and could be included into a normal diet for long lasting supplementation. The huge amount of data describing curcumin activity provided convincing evidence concerning its beneficial effects. One of them could be regulation of sirtuin level/activity. However, it has to be kept in mind that all natural compounds, including curcumin, have pleiotropic activity and many molecular and cellular targets. On the other hand, the ageing process per se is multifactorial, and modulation of sirtuin level/activity, especially in such complex organism as the human being, could not be sufficient to slow it down.
